# Risk of Psychosis Among Individuals Who Have Presented to Hospital With Self-harm: A Prospective Nationwide Register Study in Sweden

**DOI:** 10.1093/schbul/sbae002

**Published:** 2024-01-19

**Authors:** Koen Bolhuis, Laura Ghirardi, Ralf Kuja-Halkola, Ulla Lång, Martin Cederlöf, Johanna Metsala, Paul Corcoran, Karen O’Connor, Philip Dodd, Henrik Larsson, Ian Kelleher

**Affiliations:** Department of Child and Adolescent Psychiatry/Psychology, Erasmus Medical Centre-Sophia Children’s Hospital, Rotterdam, The Netherlands; Department of Psychiatry, Royal College of Surgeons in Ireland, Dublin, Ireland; Department of Medical Epidemiology and Biostatistics, Karolinska Institutet, Stockholm, Sweden; Department of Medical Epidemiology and Biostatistics, Karolinska Institutet, Stockholm, Sweden; MediNeos Observational Research—IQVIA, Data Management & Statistics, Modena, Italy; Department of Medical Epidemiology and Biostatistics, Karolinska Institutet, Stockholm, Sweden; Centre for Clinical Brain Sciences, University of Edinburgh, Edinburgh, UK; Finnish Institute for Health and Welfare, Helsinki, Finland; University of Oulu, School of Medicine, Oulu, Finland; Department of Medical Epidemiology and Biostatistics, Karolinska Institutet, Stockholm, Sweden; School of Medical Sciences, Faculty of Medicine and Health, Örebro University, Örebro, Sweden; Centre for Clinical Brain Sciences, University of Edinburgh, Edinburgh, UK; Finnish Institute for Health and Welfare, Helsinki, Finland; National Suicide Research Foundation, University College Cork, Cork, Ireland; School of Public Health, University College Cork, Cork, Ireland; Rise, South Lee Mental Health Services, Cork & Department of Psychiatry, University College Cork, Cork, Ireland; National Clinical Programme for Early Intervention in Psychosis, Health Service Executive Dublin, Dublin, Ireland; National Office for Suicide Prevention, Health Service Executive Dublin, Dublin, Ireland; Department of Medical Epidemiology and Biostatistics, Karolinska Institutet, Stockholm, Sweden; School of Medical Sciences, Faculty of Medicine and Health, Örebro University, Örebro, Sweden; Centre for Clinical Brain Sciences, University of Edinburgh, Edinburgh, UK; University of Oulu, School of Medicine, Oulu, Finland; University College Dublin, School of Medicine, Dublin, Ireland

**Keywords:** prediction, suicide, self-harm, register, schizophrenia, bipolar disorder

## Abstract

**Background and Hypothesis:**

Recent research showed that young people who presented to hospital with self-harm in Finland had a significantly elevated risk of later psychosis. We investigated the prospective relationship between hospital presentation for self-harm and risk of psychosis in an unprecedentedly large national Swedish cohort.

**Study Design:**

We used inpatient and outpatient healthcare registers to identify all individuals born between 1981 and 1993 who were alive and living in Sweden on their 12th birthday and who presented to hospital one or more times with self-harm. We compared them with a matched cohort, followed up for up to 20 years, and compared the cumulative incidence of psychotic disorders. Furthermore, we examined whether the strength of the relationship between hospital presentation for self-harm and later psychosis changed over time by examining for cohort effects.

**Study Results:**

In total, 28 908 (2.0%) individuals presented to hospital with self-harm without prior psychosis diagnosis during the follow-up. For individuals who presented to hospital with self-harm, the cumulative incidence of diagnosed psychosis was 20.7% at 20 years follow-up (hazard radio = 13.9, 95% CI 13.3–14.6, *P*-value <5 × 10^−308^). There was no evidence of a dilution of the effect over time: while the incidence of hospital self-harm presentation increased, this did not result in an attenuation over time of the strength of the relationship between hospital self-harm presentation and subsequent psychosis.

**Conclusions:**

Individuals who present to hospital with self-harm in their teens and 20s represent an important risk group for psychosis prediction and prevention.

## Introduction

A strong relationship between psychosis and self-harm has long been established.^1^ Most research to date, however, has focused on the high rates of self-harm that occur in individuals already diagnosed with a psychotic disorder.^2–5^ Recent research, on the other hand, has demonstrated that self-harm may, in some cases, precede psychosis diagnosis and therein act as a risk marker for future psychotic disorder.^6,7^ Using Finnish healthcare register data on all individuals born in 1987 (*N* = 59 476), we recently showed that young people who presented to hospital with self-harm had a greatly increased risk of later psychosis.^6^ In total, 18% of these individuals were diagnosed with a psychotic disorder by age 28, suggesting that individuals with hospital self-harm presentation might represent an important high-risk group for psychosis,^8^ alongside the clinical high-risk (CHR) approach.^9^

In the present study, we used an unprecedentedly large Swedish population cohort comprising >1.4 million individuals to investigate the relationship between hospital presentation for self-harm and subsequent risk for psychosis. What is more, given that the incidence of hospital presentation for self-harm has been increasing over time,^10–14^ we also tested whether this increase might cause a dilution over time in the absolute risk of psychosis in individuals who have presented to hospital with self-harm. In addition, we aimed to study whether the association between hospital presentation with self-harm and subsequent psychosis differed depending on age at first hospital presentation.

## Methods

### Study Population

Several national Swedish registers were combined using unique personal identification numbers.^15^ All individuals born in Sweden between 1981 and 1993, alive and living in Sweden at their 12th birthday were identified through the Total Population Register.^16^ Only individuals with identifiable biological parents were included, resulting in a final cohort sample of 1 426 537. Individuals were followed from their 12th birthday until outcome diagnosis, death, migration outside Sweden, or end of follow-up on December 31, 2013, whichever came first. Hence, the maximum potential duration of follow-up was until age 32 years. This study was approved by the Regional Ethics Committee in Stockholm, Sweden (Registration Number 2013/862–31/5). In accordance with Swedish law, the requirement for informed consent was waived as this study was register based and data were pseudonymized. Importantly, it was not possible to personally identify individuals at any time.

### Participants and Matching

Each individual who presented to hospital with self-harm without a prior diagnosis of psychosis (for more detailed case definition, see paragraph below) was matched on birth year and sex with 10 individuals without a hospital presentation with self-harm during the follow-up. The matched individuals were considered at risk for psychosis from the date of the first self-harm presentation of the exposed individuals to whom they were matched. The included individuals fulfilled the following criteria: alive at the date of matching, no emigration out of Sweden, and not diagnosed with psychosis or self-harm prior to the date of matching. Follow-up continued until psychosis diagnosis, emigration, death, or the end of follow-up in registers (December 31, 2013), whichever came first.

### Psychiatric Phenotypes

#### Hospital Presentation for Self-harm

Hospital presentations of self-harm behaviors from age 6 years onwards were identified using the National Patient Register.^16^ All hospital registrations for injury or poisoning are coded by a code for external causes of morbidity indicating the cause of the injury/poisoning.^16^ Self-harm was classified using the Swedish national modification of ICD-9 diagnostic codes E950–E959 prior to 1997, and ICD-10 diagnostic codes X60–X84 during follow-up, in line with previous work.^17^ Records from outpatient physician visits to hospitals were available from 2001 onwards.^16^ We used data on the primary and secondary diagnoses recorded as the reason for the healthcare visit, as well as from records on external reasons for physical harm (eg, “intentional self-harm by a sharp object” when the primary diagnosis was an “open wound of forearm”).

#### Outcome Diagnoses of Psychosis

Psychosis diagnoses assigned from age 12 years onwards were obtained through the National Patient Register. Psychosis diagnoses included diagnoses of affective and non-affective psychosis diagnoses, including schizophrenia and bipolar disorder. Similarly, inpatient cases were identified for the full follow-up period and outpatient registrations were available from 2001 onwards. See supplementary tables S1 and S2 for the included ICD-9 and ICD-10 codes, respectively.

### Statistical Analyses

Lifetime prevalence of hospital presentations with self-harm and psychosis was calculated for the full sample and separately for males and females.

The first aim was to assess the association between hospital presentations with self-harm and subsequent diagnoses of psychosis. First, we estimated cumulative incidence of psychosis for those with and without a hospital presentation with self-harm in order to quantify its associations with psychosis on an absolute scale. Start of the follow-up was from the time at hospital presentation with self-harm and we used Kaplan-Meier estimation to assess the cumulative incidence as 1 minus the survival function with the underlying timescale being the time since first hospital presentation with self-harm. We calculated cumulative incidence for each year until endpoint at 20 years, interpreted as the probability of psychosis occurring within 20 years, while accounting for censoring. The relative risks of the associations were quantified as hazard ratios (HRs) and their 95% CIs based on Cox proportional hazards models. Next, analyses were stratified by birth sex. No sociodemographic covariates were included in the model to comply with a predictive (ie, noncausal) approach to estimate absolute risks.

To address the second aim, we calculated the cumulative incidence for psychosis in individuals who presented to hospital for self-harm separately for birth year groupings to assess for cohort effects. As incidence of hospital presentation for self-harm has been increasing over time, we wished to investigate whether this might have a dilution effect on the strength of the relationship with psychosis risk over time. This was done for 3 groups, ie, born between 1981 and 1985, born between 1986 and 1989, and born between 1990 and 1993.

To address the third aim, we examined differential cumulative incidence of psychosis in individuals who presented to hospital before age 18 years, between ages 18 and 21 years vs after age 21 years. These age groupings were the same as in our previous study to facilitate comparison.^6^ This was calculated using data from the cohort diagnosed with self-harm in 2003, separated by abovementioned age groups, and using attained age as underlying timescale. For this analysis we allowed delayed entry, meaning that individuals contributed to follow-up from the age of self-harm (or corresponding age for those matched). This allowed follow-up time to be more similar with regard to calendar time, and also allowed for comparison at specific attained ages (rather than only time since diagnosis).

In a secondary analysis, we excluded registrations for bipolar disorder without psychotic symptoms (ie, ICD-10 codes F30.0, F30.1, F30.8, F30.9, F31.0, F31.1, F31.3, F31.4, F31.6, F31.8, and F31.9) to focus on the relationship between hospital presentation with self-harm and subsequent more specifically defined psychosis.

SAS and R were used for data management and analyses were done in R using the survival package^18,19^ and visual presentations were performed using the survminer package.^20^

## Results

### Descriptive Characteristics

In the source population of 1 426 537 individuals, hospital presentation with self-harm without prior psychosis was more common among females than males (table 1; 2.8% vs 1.3%, respectively). In the total cohort, 1.5% of individuals received a psychosis diagnosis. Lifetime prevalence of psychosis was higher in females compared with males (1.6% vs 1.3%). Of all psychosis diagnoses, *n* = 3364 (16.2%) were preceded by a hospital presentation with self-harm.

**Table 1.** Descriptive Characteristics of the Sample

	Overall	Males	Females
	*N* (%)	*N* (%)	*N* (%)
Sample size	1 426 537	733 696 (51.4)	692 841 (48.6)
Hospital presentation with self-harm^a^	28 908 (2.0)	9609 (1.3)	19 299 (2.8)
Before age 18 years^b^	10 877 (37.6)	2488 (25.9)	8389 (43.5)
Between ages 18 and 21 years^b^	8557 (29.6)	2865 (29.8)	5692 (29.5)
After age 21 years^b^	9474 (32.8)	4256 (44.2)	5218 (27.0)
Psychosis diagnosis	20 717 (1.5)	9650 (1.3)	11 067 (1.6)
Psychosis preceded by self-harm^c^	3364 (16.2)	975 (10.1)	2389 (21.6)

^a^Excluding individuals with psychosis prior to self-harm.

^b^Percentages presented here represent the individuals with a first hospital presentation with self-harm in the respective age category divided by the total number individuals with hospital presentations with self-harm.

^c^Percentage refers to proportion of all individuals with psychosis diagnosis that were preceded by hospital presentations with self-harm.

### Prospective Associations Between Hospital Presentation With Self-harm and Subsequent Psychosis Diagnoses

**Fig. 1.**
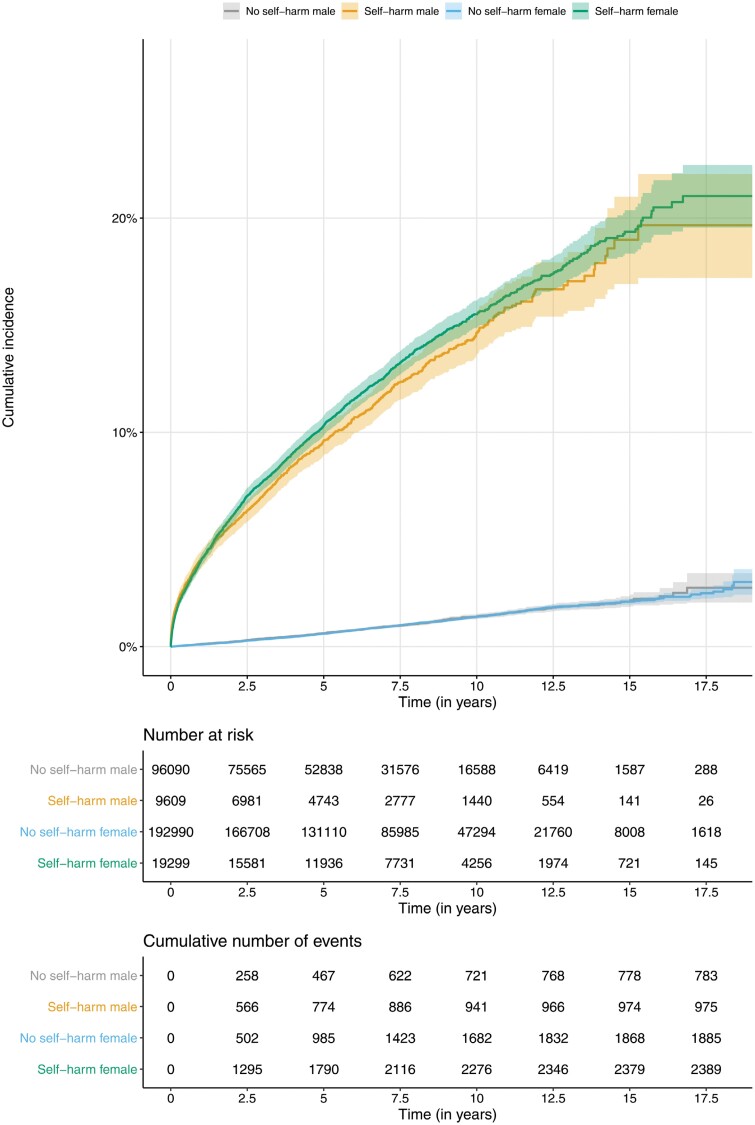
Kaplan-Meier cumulative incidence curves for psychosis separated by history of hospital presentation for self-harm.

The cumulative incidence of psychosis was increased for individuals who presented to hospital following self-harm compared with those who did not (figure 1 and supplementary table S3). For the total 20 years of follow-up time, the cumulative incidence was 20.7% (95% CI 19.4–22.0) for individuals who presented to hospital with self-harm vs 3.0% (95% CI 2.5–3.4) for those who did not. This corresponded to a HR of 13.9 (95% CI 13.3–14.6, *P*-value <5 × 10^−308^). The 5-, 10-, and 15-year cumulative incidences for individuals who presented to hospital with self-harm vs the matched comparison group were 10.1% vs 0.6% (HR = 35.5, 95% CI 33.4–37.7); 15.3% vs 1.4% (HR = 8.1, 95% CI 7.5–8.8); and 19.2% vs 2.1% (HR = 3.5, 95% CI 3.0–4.0), respectively. Figure 1 and supplementary table S4 indicate that cumulative incidence rates were comparable for males and females (HR = 1.1, 95% CI 1.0–1.1, *P*-value = .128).

### Change in the Relationship Between Hospital Presentation for Self-harm and Psychosis Risk Over Time

**Fig. 2.**
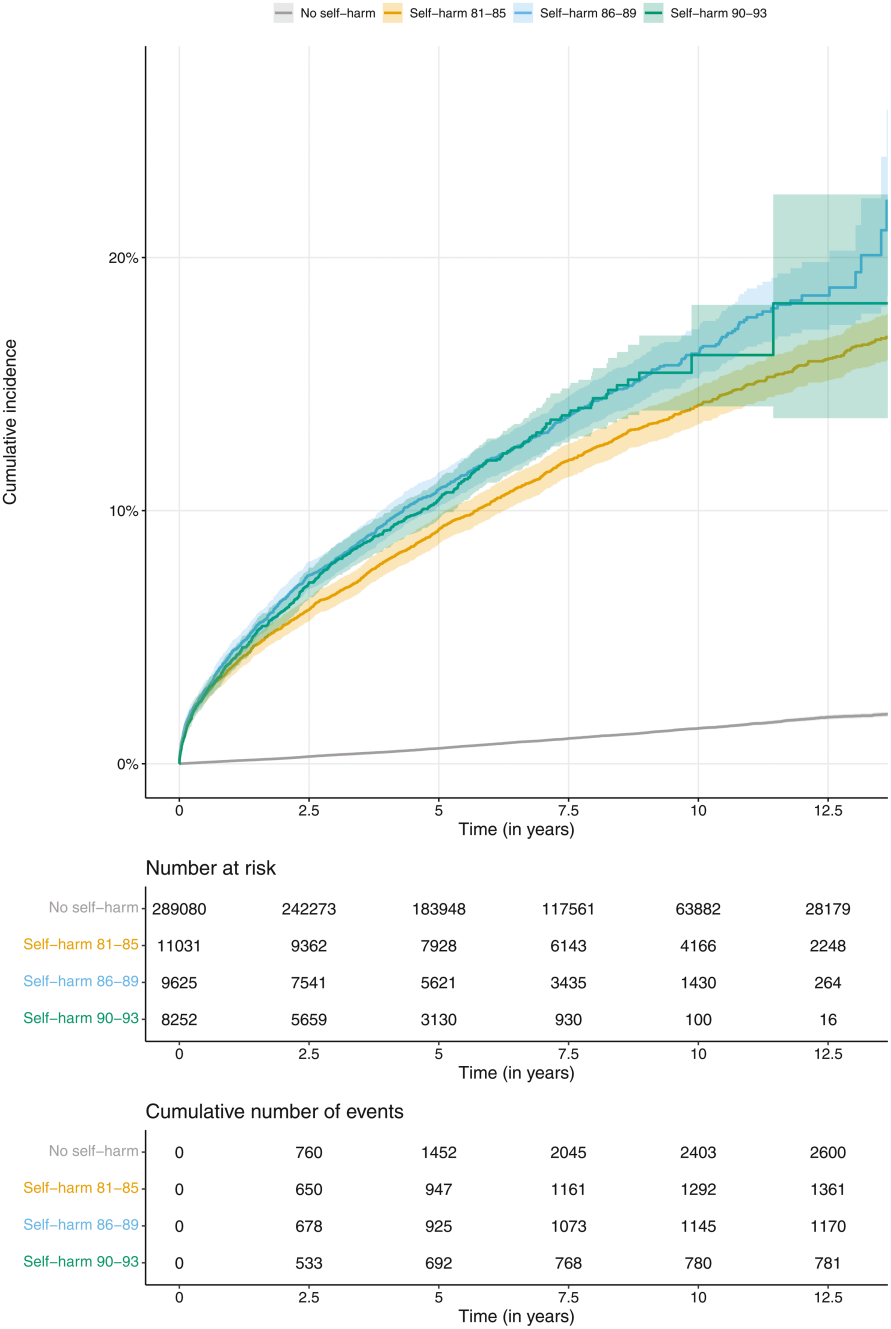
Kaplan-Meier cumulative incidence curves for psychosis separated by history of hospital presentation for self-harm and separately for 3 different birth cohorts.

In order to assess the association between hospital presentation for self-harm and psychosis over time, we looked at 3 separate subcohorts within the overall sample: a birth cohort including all individuals born between 1981 and 1985, between 1986 and 1989, and between 1990 and 1993 (figure 2 and supplementary table S5). The cumulative incidence and HR were slightly lower for the older 1981–1985 cohort (HR = 12.1, 95% CI 11.3–12.9) compared with the younger 1986–1989 (HR = 14.7, 95% CI 13.6–15.9) and 1990–1993 (HR = 17.2, 95% CI 15.6–19.1) cohorts. Differences were small but statistically significant (*P* = 1.31 × 10^−8^). Of note, CIs were particularly wide for the younger cohort due to lower numbers of individuals at risk and, correspondingly, lower numbers of events compared with the older 1981–1985 cohort.

### Exclusion of Bipolar Affective Disorder

After exclusion of bipolar affective disorder without psychotic symptoms classifications, the 20-year cumulative incidence for more specifically defined psychosis in individuals who presented to hospital after self-harm was 10.0% (95% CI 9.1%–10.9%, supplementary figure S1) vs 1.2% (95% CI 1.0%–1.4%) for nonexposed individuals, corresponding to a HR = 13.4 (95% CI 12.6–14.3, *P* < 5 × 10^−308^). Cumulative incidence was higher for males vs females (13.8%, 95% CI 11.7–15.7 vs 8.7%, 95% CI 7.7–9.6, respectively, but relative risks were similar (HR_male_ = 12.8, 95% CI 11.6–14.1; HR_female_ = 13.9, 95% CI 12.8–15.0, *P*_difference_ = 0.203). Of all these psychosis registrations, *n* = 1736 (14.4%) were preceded by a hospital presentation with self-harm.

### Differential Risk for Age at First Hospital Presentation With Self-harm

**Fig. 3.**
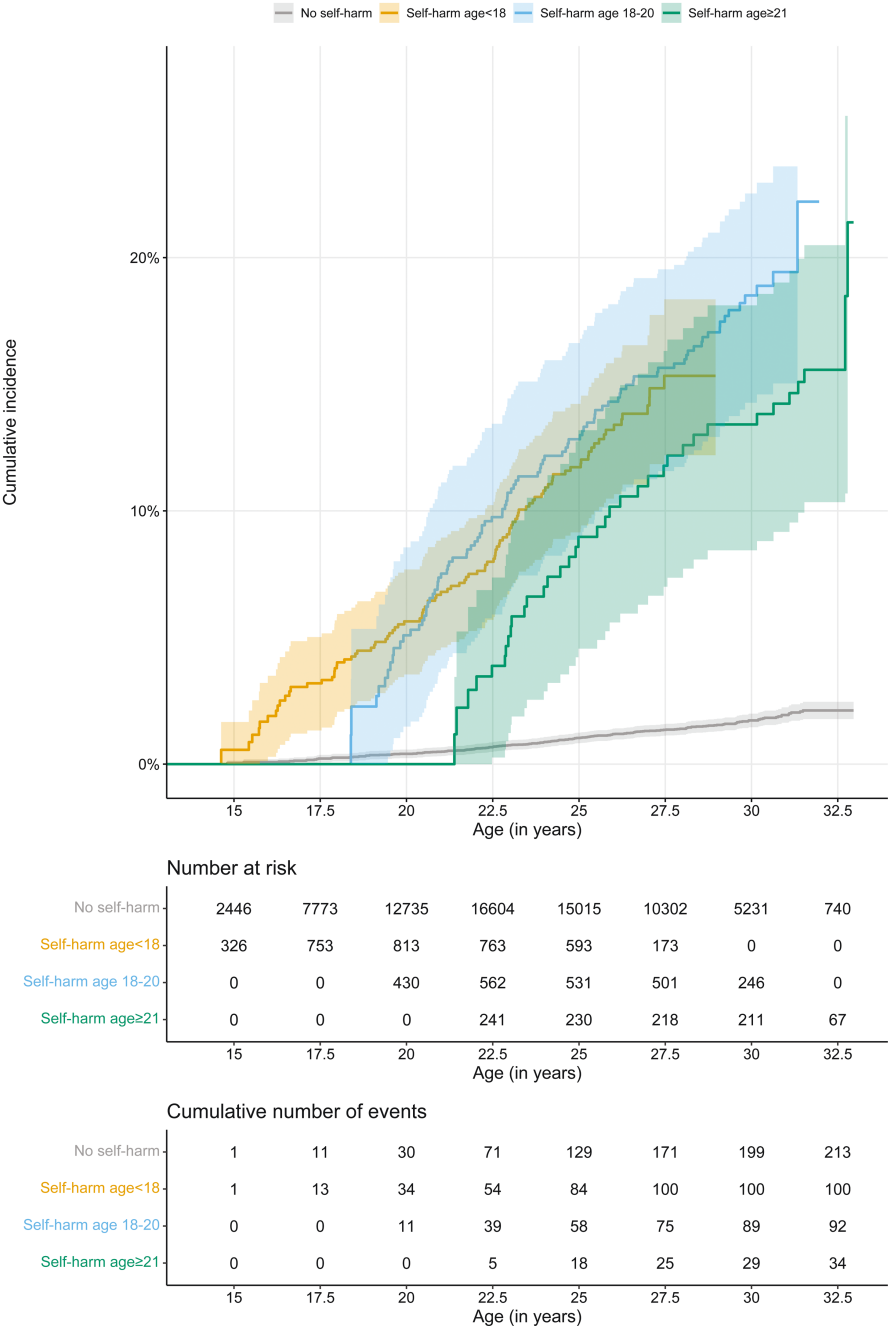
Kaplan-Meier cumulative incidence curves for psychosis separated by age at first of hospital presentation for self-harm, with age as the underlying timescale.

In the cohort who all had their first hospital presentation with self-harm in 2003, cumulative incidence for psychosis outcomes were similar between the groups who presented to hospital before age 18 years, between ages 18 and 21 years, or after age 21 years, as 95% CIs were overlapping (figure 3 and supplementary table S6). More specifically, at age 27.5 years, the cumulative incidence of psychosis for the 3 age groups were 15.3% (95% CI 12.2%–18.4%; HR = 11.4, 95% CI 8.9–14.6) for the self-harm before age 18 years group, 15.6% (95% CI 11.5%–19.5%; HR = 13.2, 95% CI 10.1–17.3) for the self-harm between ages 18 and 21 years group, and 11.8% (95% CI 7.0%–16.3%; HR = 11.9, 95% CI 7.9–18.1) for the self-harm after age 21 years group, which did not differ statistically significantly between the age-of-onset groups (*P*-value age <18 against age 18–21 years = 0.902, *P*-value age <18 against age ≥21 years = 0.213, and *P*-value age 18–21 against age ≥21 years = 0.217). Similarly, at the earlier ages of follow-up (ie, 22.5, and 25 years), CIs were overlapping between the 3 age-at-first-self-harm groups. The older age groups showed a steeper rise in number of psychoses following hospital presentation with self-harm compared with the youngest cohort who presented to hospital before age 18 years (figure 3).

## Discussion

In the largest study of its kind, we found that 21% of all individuals who had presented to a Swedish hospital with self-harm went on to be diagnosed with a psychotic disorder by the study endpoint. This level of risk was comparable to that of individuals diagnosed with a CHR syndrome,^21^ suggesting that this group represents an important additional high-risk cohort for the prediction and prevention of severe mental illness. In addition, we found that cumulative risk for psychosis was similar for those who presented to hospital with self-harm at different ages, ie, before age 18 years, between ages 18 and 21 years, and age 21 years or older. Of all individuals who were diagnosed with psychosis throughout the follow-up, 16% had an earlier hospital presentation for self-harm injuries before being diagnosed with psychosis.

In the case of approximately half of our outcome diagnoses, the diagnosis fell into the category of bipolar affective disorder without psychotic symptoms, demonstrating the importance of considering bipolar disorder as one of the severe mental disorder outcomes predicted by presentation to hospital with self-harm in youth, with or without psychotic symptoms. Overall, looking only at more narrowly defined psychosis (ie, excluding bipolar affective disorder without psychotic symptoms), males who presented to hospital with self-harm were more likely to have subsequent psychosis (13.8%) than females (8.7%) by the study endpoint.

Although the incidence of self-harm has been increasing over time,^10–14,22^ we found no evidence that this increase was leading to a reduction in the strength of the relationship with psychosis risk. On the contrary, the relative risk of psychosis was stronger for the younger birth cohorts. This suggests that increased hospital presentations for self-harm over time, rather than resulting in a dilution of absolute risk, may, in fact, be resulting in an increased capture of individuals at risk of psychosis. The findings will need to be further investigated using more recent healthcare register data.

It is important to emphasize that the elevated psychosis risk demonstrated in the current study does not relate to self-harm ideations or behaviors per se, but rather relates to contact with a specific clinical pathway (ie, hospital presentation for self-harm). Only a small proportion of cases of self-harm present to hospital and so it is important that the current results are not extrapolated to a broad self-harm phenotype in the general population. One recent study looked at the relationship between thoughts of self-harm in a general population cohort and subsequent risk for psychosis. Young people who reported thoughts of self-harm at age 17 had a 7-fold increased odds of later psychotic disorder by age 24, compared with young people who did not report thoughts of self-harm.^23^ The absolute risk of psychosis in this group, however, was just 3%, demonstrating that self-harm or thoughts of self-harm, in and of themselves, could not be considered markers of high psychosis risk. Rather, it is factors associated with hospital presentation for self-harm that are associated with high psychosis risk.

There are many differences between self-harm at a population level vs in the context of hospital presentations, including the severity of injury, motivating factors for self-harm, associated levels of distress, and responses to self-harm incidents by caregivers.^24,25^ All of these factors (and many more) may explain the reasons for the relationship between hospital presentation for self-harm and elevated psychosis risk. As these factors are not covered by Swedish healthcare registers, further research will be needed to clarify the specific causal mechanisms but our findings show that, whatever the underlying causal factors, individuals who make contact with this clinical pathway represent an important group for future psychosis prediction and prevention efforts.

We conducted sensitivity analyses on subsamples diagnosed with self-harm between the years 1981 and 1985, 1986 and 1989, and 1990 and 1993 because with advancing time the quality of the outpatient registers and use of ICD-10 codes improved significantly.^15,16^ These sensitivity analyses demonstrated that the association between hospital presentation with self-harm and subsequent psychosis was similar across the 3 birth cohorts. At the same time, it is important to note that the prevalence of psychotic disorder diagnoses in Swedish healthcare registers was substantially lower than we previously found in Finland (0.9% vs 3.2%),^6^ even though follow-up was longer in the Swedish sample. This is unlikely to be due to a true difference in the prevalence of psychosis between the 2 countries, as demonstrated by the Global Burden of Disease study.^26^ Rather, the Swedish outpatient register started in 2001 and may have been relatively incomplete for the first number of years, perhaps resulting in underreporting of psychosis and bipolar disorder cases. It could also be the case that psychotic disorders are underdiagnosed in Swedish clinical services. Alternatively, one might consider that psychotic disorders are overdiagnosed in Finnish clinical services but research suggests high validity for register diagnoses of psychotic disorders in Finland,^27^ making this explanation unlikely. Overall, this suggests that any bias in our findings would be toward an underestimation, rather than an overestimation, of the risk of psychosis in young people who present to hospital with self-harm.

The absolute risk for psychosis associated with hospital presentation for self-harm was comparable to the level of risk associated with a formal diagnosis of a CHR syndrome for psychosis.^21,28–30^ Individuals with CHR syndromes typically receive specialist diagnostic psychiatric assessment and up to 3 years of follow-up in specialized mental health services. In contrast, hospital presentation for self-harm in young people is typically formulated as a “psychosocial crisis” or an emerging personality disorder, with little consideration given to the possibility of risk for psychosis. The similar level of psychosis risk associated with hospital presentation for self-harm compared with formal CHR diagnosis suggests that this population should be afforded the same opportunities for psychiatric follow-up, such as the long-term follow-up and multidisciplinary diagnostic assessment.

A psychosocial crisis/personality disorder formulation is especially frequently applied to girls and young women who present to hospital with self-harm.^31^ We found, however, that the absolute risk of our broad psychosis outcome was just as elevated in females who presented to hospital with self-harm as it was in males, although more specifically defined psychosis was more prevalent in males. Beyond that, the proportion of future broad psychosis cases captured by this clinical pathway was substantially higher for females than for males: in total, 22% of all subsequent female psychosis diagnoses were preceded by hospital presentation with self-harm, compared with 10% of all male psychosis diagnoses. This suggests that this clinical pathway presents particularly strong opportunities for psychosis prediction and prevention in girls and young women. It also highlights the importance of avoiding overly simplistic or reductionist interpretations of the clinical significance of hospital presentation for self-harm in girls and young women.^31–34^

To improve prediction and early intervention of psychosis onset, multimodal and multi-setting prediction approaches are needed.^35,36^ These need to be implemented in a sequential stepwise manner to improve acceptability and feasibility.^35^ Clinical systems pathways, such as the hospital setting as discussed in this study, can be considered an important step herein as identification of risk is based on data that are already routinely collected (ie, do not require additional costly assessments). Future research to further improve personalized risk prediction for psychosis following hospital presentation with self-harm will need to take additional factors into account, such as contact with child and adolescent mental health services (CAMHS),^37^ minority stress and social disadvantage,^38^ family history of mental disorders.^39^ Additional clinical, demographic, cognitive, and biological markers may also help to stratify risk for psychosis in this group. Further research will be needed to explore this. Furthermore, considering the results from our secondary analysis focusing on more narrowly defined psychosis registrations, careful assessment of (prodromal) mood dysregulation may be of added value in determining risk.^40,41^

Strengths of the current study include its large size, whole-population coverage, and lack of attrition due to the use of healthcare registers. This study replicates and extends previous findings using Finnish register data,^6^ which is important both from a theoretical perspective, considering the “replication crisis” in psychological sciences,^42,43^ and from a clinical perspective, considering the need to increase our ability to identify people at risk of psychosis. We have been able to investigate the magnitude of the association between hospital presentation with self-harm and subsequent psychosis over time, but future studies with more recent healthcare registration data should extend this into more recent times. A limitation is that, as discussed in more detail above, diagnoses of psychotic disorders may be under-recorded in Swedish outpatient register data, especially in earlier years of the register.

## Conclusion

Individuals who present to hospital with self-harm are a high-risk group for subsequent psychotic disorder diagnosis: in total, 21% were diagnosed with a psychotic disorder by the study endpoint. These findings highlight important opportunities for earlier identification of risk for severe mental disorders and open new avenues for research into psychosis prevention.

## Supplementary Material

Supplementary material is available at https://academic.oup.com/schizophreniabulletin/.

sbae002_suppl_Supplementary_Tables_S1-S6_Figures_S1

## Data Availability

Data came from a linkage of Swedish registers. Data were accessed and analyzed at the Department of Medical Epidemiology and Biostatistics at Karolinska Institutet, Sweden. Requests for study protocols and analysis plans can be made through contacting the first and last authors (K.B. and I.K.).
