# Supplementary material for: Risk of Psychosis Among Individuals Who Have Presented to Hospital With Self-harm: A Prospective Nationwide Register Study in Sweden
Source: Schizophr Bull. 2024 Jan 19;50(4):881–90. doi: 10.1093/schbul/sbae002 (PMC11283185; doi:10.1093/schbul/sbae002)

**Supplemental material**

**Table S1**: ICD-9 codes included in the psychosis outcome.

| **Disorders** | **ICD-9 labels** | **ICD-9 codes**  **international** | **ICD-9 codes**  **Swedish** |
| --- | --- | --- | --- |
| Schizophrenia | Simple type | 295.0 | 295A |
|  | Hebephrenic type | 295.1 | 295B |
|  | Catatonic type | 295.2 | 295C |
|  | Paranoid type | 295.3 | 295D |
|  | Acute schizophrenic episode | 295.4 | 295E |
|  | Residual schizophrenia | 295.6 | 295G |
|  | Other specified types | 295.8 | 295W |
|  | Unspecified schizophrenia | 295.9 | 295X |
| Other non-affective psychoses | Paranoia | 297.1 | 297B |
|  | Induced psychosis | 297.3 | 297D |
|  | Other and unspecified reactive psychosis | 298.8 | 298W |
|  | Unspecified psychosis | 298.9 | 298X |
|  | Schizoaffective disorder | 295.7 | 295H |
| Affective Psychoses | Manic-depressive psychosis, depressed type | 296.1 | 296B |
|  | Manic-depressive psychosis, manic type | 296.0 | 296A |
|  | Manic-depressive psychosis, circular type but currently manic | 296.2 | 296C |
|  | Manic-depressive psychosis, circular type but currently depressed | 296.3 | 296D |
|  | Manic-depressive psychosis, circular type, mixed | 296.4 | 296E |
|  | Other affective psychoses | 296.8 | 296W |
|  | Unspecified affective psychoses | 296.9 | 296X |
| Bipolar disorder without psychotic symptoms | NA | NA | NA |

**Table S2:** ICD-10 codes included in the psychosis outcome.

| **Disorders** | **ICD-10 labels** | **ICD-10 codes** |
| --- | --- | --- |
| Schizophrenia | Paranoid schizophrenia | F20.0 |
|  | Hebephrenic schizophrenia | F20.1 |
|  | Catatonic schizophrenia | F20.2 |
|  | Undifferentiated schizophrenia | F20.3 |
|  | Post-schizophrenic depression | F20.4 |
|  | Residual schizophrenia | F20.5 |
|  | Simple schizophrenia | F20.6 |
|  | Other schizophrenia | F20.8 |
|  | Schizophrenia, unspecified | F20.9 |
| Other non-affective psychoses | Persistent delusional disorders | F22 |
|  | Acute and transient psychotic disorders | F23 |
|  | Induced delusional disorder | F24 |
|  | Schizoaffective disorders | F25 |
|  | Other nonorganic psychotic disorders | F28 |
|  | Unspecified nonorganic psychosis | F29 |
| Affective Psychoses | Mania with psychotic symptoms | F30.2 |
|  | Bipolar affective disorder, current episode manic with psychotic symptoms | F31.2 |
|  | Bipolar affective disorder, current episode severe depression with psychotic symptoms | F31.5 |
|  | Severe depressive episode with psychotic symptoms | F32.3 |
|  | Recurrent depressive disorder, current episode severe with psychotic symptoms | F33.3 |
| Bipolar Disorder without psychotic symptoms | Hypomania | F30.0 |
|  | Mania without psychotic symptoms | F30.1 |
|  | Other manic episodes | F30.8 |
|  | Manic episode, unspecified | F30.9 |
|  | Bipolar affective disorder, current episode hypomanic | F31.0 |
|  | Bipolar affective disorder, current episode manic without psychotic symptoms | F31.1 |
|  | Bipolar affective disorder, current episode mild or moderate depression | F31.3 |
|  | Bipolar affective disorder, current episode severe depression without psychotic symptoms | F31.4 |
|  | Bipolar affective disorder, current episode mixed | F31.6 |
|  | Bipolar affective disorder, currently in remission | F31.7 |
|  | Other bipolar affective disorders | F31.8 |
|  | Bipolar affective disorder, unspecified | F31.9 |
| Bipolar Disorder with psychotic symptoms | Mania with psychotic symptoms | F30.2 |
|  | Bipolar affective disorder, current episode manic with psychotic symptoms | F31.2 |
|  | Bipolar affective disorder, current episode severe depression with psychotic symptoms | F31.5 |

**Table S3**: Cumulative incidence (95% confidence interval) of any psychotic disorder for the full cohort separated on hospital presentation with self-harm.

| **Time since self-harm** | **1 years** | **2 years** | **3 years** | **4 years** | **5 years** |
| --- | --- | --- | --- | --- | --- |
| No self-harm | 0.1% (0.1-0.1) | 0.2% (0.2-0.2) | 0.3% (0.3-0.4) | 0.5% (0.4-0.5) | 0.6% (0.6-0.6) |
| Self-harm | 4.1% (3.8-4.3) | 6.0% (5.7-6.2) | 7.5% (7.2-7.8) | 8.9% (8.5-9.2) | 10.1% (9.7-10.5) |
| **Time since self-harm** | **6 years** | **7 years** | **8 years** | **9 years** | **10 years** |
| No self-harm | 0.8% (0.7-0.8) | 0.9% (0.9-1.0) | 1.1% (1.0-1.1) | 1.2% (1.2-1.3) | 1.4% (1.3-1.5) |
| Self-harm | 11.3% (10.9-11.7) | 12.3% (11.9-12.8) | 13.5% (13.1-14.0) | 14.4% (13.9-14.9) | 15.3% (14.7-15.8) |
| **Time since self-harm** | **11 years** | **12 years** | **13 years** | **14 years** | **15 years** |
| No self-harm | 1.6% (1.5-1.6) | 1.8% (1.7-1.8) | 1.9% (1.8-2.0) | 2.0% (1.9-2.1) | 2.1% (2.0-2.2) |
| Self-harm | 16.2% (15.6-16.8) | 17.0% (16.3-17.6) | 17.6% (16.9-18.3) | 18.6% (17.8-19.4) | 19.2% (18.3-20.1) |
| **Time since self-harm** | **16 years** | **17 years** | **18 years** | **19 years** | **20 years** |
| No self-harm | 2.3% (2.1-2.4) | 2.5% (2.2-2.7) | 2.6% (2.3-2.9) | 3.0% (2.5-3.4) | 3.0% (2.5-3.4) |
| Self-harm | 20.3% (19.1-21.4) | 20.7% (19.4-22.0) | 20.7% (19.4-22.0) | 20.7% (19.4-22.0) | 20.7% (19.4-22.0) |

**Table S4**: Cumulative incidence (95% confidence interval) of any psychotic disorder separately for males and females.

| **Time since self-harm** | **1 years** | **5 years** | **10 years** | **15 years** |  |
| --- | --- | --- | --- | --- | --- |
| No self-harm male | 0.1% (0.1-0.1) | 0.6% (0.6-0.7) | 1.4% (1.3-1.5) | 2.1% (1.9-2.3) |  |
| Self-harm male | 4.1% (3.7-4.5) | 9.6% (9.0-10.3) | 14.6% (13.6-15.6) | 19.0% (16.9-21.0) |  |
|  |  |  |  |  |  |
| No self-harm female | 0.1% (0.1-0.1) | 0.6% (0.6-0.6) | 1.4% (1.3-1.5) | 2.1% (2.0-2.2) |  |
| Self-harm female | 4.0% (3.8-4.3) | 10.3% (9.9-10.8) | 15.5% (14.9-16.1) | 19.4% (18.3-20.4) |  |

**Table S5**: Cumulative incidence (95% confidence interval) of any psychotic disorder for the three birth cohorts separated on hospital presentation with self-harm.

| **Time since self-harm** | 1 years | **5 years** | **10 years** | **15 years** |
| --- | --- | --- | --- | --- |
| No self-harm born 1981-1985 | 0.1% (0.1-0.1) | 0.6% (0.5-0.6) | 1.3% (1.2-1.4) | 2.0% (1.9-2.1) |
| Self-harm born 1981-1985 | 3.8% (3.4-4.2) | 9.3% (8.7-9.8) | 14.2% (13.4-14.9) | 17.9% (16.9-18.9) |
|  |  |  |  |  |
| No self-harm born 1986-1989 | 0.1% (0.1-0.1) | 0.6% (0.6-0.7) | 1.5% (1.4-1.6) | 2.5% (1.6-3.4) |
| Self-harm born 1986-1989 | 4.4% (3.9-4.8) | 10.8% (10.2-11.5) | 16.2% (15.2-17.2) | 22.2% (18.5-25.8) |
|  |  |  |  |  |
| No self-harm born 1990-1993 | 0.1% (0.1-0.1) | 0.7% (0.6-0.7) | 1.5% (1.2-1.7) | 2.2% (0.9-3.4) |
| Self-harm born 1990-1993 | 4.0% (3.6-4.5) | 10.4% (9.7-11.2) | 16.1% (14.1-18.1) | 18.2% (13.7-22.5) |

**Table S6**: Cumulative incidence (95% confidence interval) of any psychotic disorder for cohort diagnosed with self-harm in 2003 for different ages of onset. Attained age as time scale, individuals has delayed enter into at-risk group.

| Time since self-harm | Age 15 | Age 17.5 | Age 20 | Age 22.5 | Age 25 | Age 27.5 |
| --- | --- | --- | --- | --- | --- | --- |
| No self-harm | 0.0%  (0.0-0.1) | 0.2%  (0.1-0.4) | 0.4%  (0.2-0.6) | 0.7%  (0.5-0.9) | 1.0%  (0.8-1.2) | 1.4%  (1.1-1.6) |
| Self-harm age <18 | 0.6%  (0.0-1.7) | 3.2%  (1.3-5.0) | 5.6%  (3.5-7.7) | 8.0%  (5.7-10.2) | 11.7%  (9.2-14.2) | 15.3% (12.2-18.4) |
| Self-harm age 18-20 | NA | NA | 5.1%  (1.5-8.6) | 9.8%  (5.9-13.5) | 12.8%  (8.8-16.6) | 15.6% (11.5-19.5) |
| Self-harm age ≥21 | NA | NA | NA | 3.9%  (0.2-7.4) | 9.0%  (4.5-13.2) | 11.8% (7.0-16.3) |

**Supplemental Figure S1**: Kaplan-Meier cumulative incidence curves for psychosis separated by history of hospital presentation for self-harm, excluding non-psychotic bipolar affective disorders.


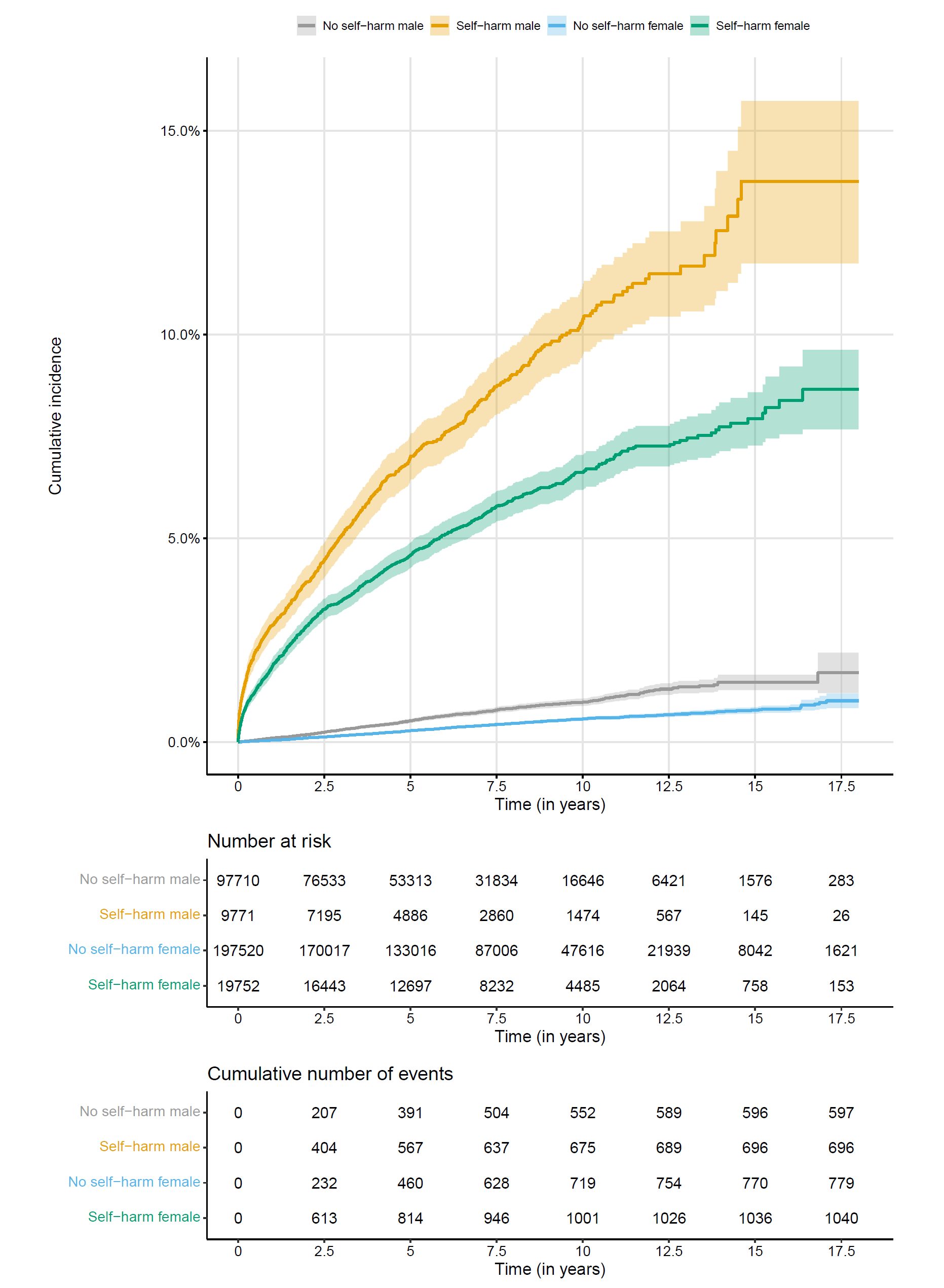

Supplement: sbae002_suppl_Supplementary_Tables_S1-S6_Figures_S1 [file sbae002_suppl_supplementary_tables_s1-s6_figures_s1.docx]
